# Metabolism rather than obesity is associated with ischemic stroke: a cross-sectional study in rural Northeastern China

**DOI:** 10.1186/s40064-016-3088-2

**Published:** 2016-08-25

**Authors:** Zhao Li, Xiaofan Guo, Yamin Liu, Naijin Zhang, Ye Chang, Yintao Chen, Yingxian Sun, Maria Roselle Abraham

**Affiliations:** 1 Department of Cardiology, The First Hospital of China Medical University, 155 Nanjing North Street, Heping District, Shenyang, 110001 Liaoning People’s Republic of China; 2 Department of Pharmacy, Zhongda Hospital, Southeast University, Nanjing, Jiangsu People’s Republic of China; 3 Department of Cardiology, Johns Hopkins University, Baltimore, MD USA

**Keywords:** Metabolically healthy obesity, Ischemic stroke, Prevalence, Risk factors

## Abstract

Little is known about stroke with different obesity phenotype as determined using the Adult Treatment Panel-III criteria with metabolic health or not. This study aimed to investigate the effects of metabolically healthy and unhealthy obesity on ischemic stroke in a general population. A total of 11,150 adults were examined using a multi-stage cluster sampling method to select a representative sample of individuals 35 years or older. Ischemic stroke was defined as history of a cerebrovascular event, as documented by doctors via either cranial CT or MR scan within the past 2 years. All subjects were categorized as having metabolically healthy non-obesity (MHNO), metabolically unhealthy non-obesity (MUNO), metabolically healthy obesity (MHO) or metabolically unhealthy obesity (MUO) using the Adult Treatment Panel-III criteria. Stratified analysis were done based on different body mass index group. For the total population, multiple regression analyses revealed that individuals with MUNO and MUO were more likely to experience ischemic stroke compared with those with MHNO (OR 2.136, 95 % CI 1.677–2.720; OR 2.712, 95 % CI 1.798–4.092; all *p* < 0.001). The OR for ischemic stroke did not significantly differ between MHO and MHNO. Stratification based on different BMI group showed that, compared with people who were normal weight without Mes, participants who were in Mes with overweight or obesity had significantly higher OR for ischemic stroke(both *p* < 0.05); participants who were not in Mes with overweight or obesity did not showed OR significantly higher. Ischemic stroke is likely associated with poor metabolic health rather than with obesity itself.

## Background

Obesity has been described as a major risk factor for metabolic complications, such as atherosclerosis and cardiovascular disease (CVD), as well as stroke (Van Gaal and Maggioni 2014; Danaei 2014; Mahmood et al. 2014). Recently, a subset of obese individuals has been identified as metabolically healthy (MHO) (Yang et al. 2015). Several studies have described MHO was an obese phenotype that does not have the burden of metabolic disorder (Boonchaya-anant and Apovian 2014; Engla 2010; Succurro et al. 2008; Chen et al. 2016). Based on this concept, metabolically unhealthy non-obesity (MUNO) and metabolically unhealthy obesity (MUO) have been identified using the Adult Treatment Panel-III (ATP-III) criteria (Chen et al. 2016; Geetha et al. 2011).

MHO has been found to be a lower risk factor for coronary artery calcification, chronic kidney disease and heart failure (Chang et al. 2014; Hosny et al. 2014). Little is known about the effects of metabolically healthy and unhealthy obesity on stroke. Therefore, the objective of this study was to determine the relation of different obesity phenotype to ischemic stroke in a general population with the hypothesis different phenotype has different influence on stroke.

## Methods

### Study population and sampling strategy

The methods used in this study have been previously published (Li et al. 2014). This study was conducted between January and August 2013 using a representative sample of individuals residing in rural areas of Liaoning Province located in Northeast China. This study adopted a multistage, stratified, random cluster sampling scheme. During the first stage, three counties (Dawa, Zhangwu, and Liaoyang) were selected from the eastern, southern, and northern regions of Liaoning Province, respectively. During the second stage, one town was randomly selected from each county (for a total of three towns). Finally, during the third stage, 6–8 rural villages from each town were randomly selected (a total of 26 rural villages were included). Participants that were pregnant or had a malignant tumor or mental disorder were excluded from the study. All eligible permanent residents aged ≥ 35 years from each village were invited to participate in the study (a total of 14,016 participants). Of them, 11,956 participants completed the study (85.3 % response rate). This study was approved by the Ethics Committee of China Medical University (Shenyang, China). All procedures were performed in accordance with ethical standards. Written consent was obtained from all participants after they were informed of the objectives, benefits, medical information and confidentiality agreement of personal information. For participants who were illiterate, we obtained written informed consent from their proxies. Among the 11,956 participants, 706 were excluded from the analysis due to incomplete data. We used baseline data, and only participants with complete data on the variables analyzed in the study were included, resulting in a final sample size of 11,150 (5115 men and 6035 women).

### Measurements and definitions

The data collection and measurement methods used in this study have been described previously (Yu et al. 2014; Chang et al. 2016).

Information on covariates, such as demographic characteristics, lifestyle risk factors, annual income and family history of chronic diseases, was collected during a single clinic visit by cardiologists and trained nurses through a face-to-face interview using a standard questionnaire. Before the survey was performed, all eligible investigators attended an organized training session that included the following subjects: the purpose of this study, the manner in which the questionnaire should be administered, the importance of standardization, the standard method of measurement, and the study procedures. After training, a strict test was used for evaluation, and only those who scored perfectly on the test became investigators. During the data collection process, a central steering committee in addition to a subcommittee for quality control ensured that all data were collected according to well-known standards.

Race was categorized as Han or other, which included some ethnic minorities in China, such as Mongol and Manchu. Educational level was divided into three categories, including primary school or below, middle school and high school or above. Self-reported sleep duration, which included nocturnal sleep and napping, was obtained from the questionnaire and was categorized as follows: ≤7, 7–8, 8–9 and >9 h/d. Annual income was categorized into three groups: ≤5000, 5000–20,000 and >20,000 CNY/year. Current drinking was defined as one or more alcoholic drinks in the previous year. Current smoking was defined as a history of 90 or more cigarettes and continued use.

Physical activities, which included occupational and leisure-time physical activities, were evaluated according to detailed descriptions (Hu et al. 2010). Occupational and leisure-time physical activities were merged and regrouped into the following 3 categories: (1) low, including light levels of both occupational and leisure-time physical activities; (2) moderate, including a moderate or high level of either occupational or leisure-time physical activity; and (3) high, including moderate or high levels of both occupational and leisure-time physical activities (De Backer and De 2004).

The body mass index (BMI) was calculated as weight in kilograms divided by the square of the height in meters. According to the American Heart Association guidelines, blood pressure (BP), which was measured three times at two-min intervals after more than 10 min of rest, was measured using a standardized automatic electronic sphygmomanometer (HEM-907; Omron, Japan). Caffeinated beverages and exercise were avoided for at least 30 min before the measurements were performed. The participants were seated appropriately during the measurements with the arm supported at the level of the heart. The mean of three blood pressure (BP) measurements was used in all analyses. Hypertension was defined as systolic blood pressure (SBP) ≥ 140 mmHg and/or diastolic blood pressure (DBP) ≥ 90 mmHg and/or current use of antihypertensive medications, according to the JNC-7 report guidelines (Bakris et al. 2015).

Fasting blood samples, which were obtained in the morning after at least 12 h of fasting, were collected from the antecubital vein into Vacutainer tubes containing EDTA. Enzymatic reactions were used to analyze the blood samples, including measurements of the fasting plasma glucose (FPG), high-density lipoprotein cholesterol (HDL-C), low-density lipoprotein cholesterol (LDL-C), triglyceride (TG), and total cholesterol (TC) levels and other routine blood biochemical indexes, with an autoanalyzer. All laboratory equipment was calibrated, and blinded duplicate samples were used in our study.

### Definitions

We used standard operating protocols to measure ATP-III components to define metabolic status (Chen et al. 2016; Manu et al. 2012), and the cut-off point for waist circumference was determined based on the WHO recommendations for the Asian population (Mi et al. 2015). Participants with 3 or more of these criteria were considered to have unhealthy metabolism (metabolic syndrome, Mes); subjects who were excluded from having unhealthy metabolism were defined as “metabolically healthy” (1) BP ≥ 130/85 mmHg and/or current use of antihypertensive medication; (2) WC ≥ 90 cm for men and ≥80 cm for women; (3) fasting glucose level ≥ 5.6 mmol/L and/or current use of antihyperglycemic medication; (4) HDL-C < 1.0 mmol/L for men and <1.3 mmol/L for women; and (5) serum TGs ≥ 1.7 mmol/L.

A BMI ≥ 28 kg/m^2^ was defined as obesity, as recommended for Asians by WHO experts (Mi et al. 2015), and a BMI < 28 kg/m^2^ was defined as non-obesity. We used this definition alongside data on Mes to create four phenotypes: metabolically healthy non-obesity (MHNO), metabolically unhealthy non-obesity (MUNO), metabolically healthy obesity (MHO) or metabolically unhealthy obesity (MUO).

For the stratification analysis based on different BMI group, consistent with the lower cut-off values recommended by WHO experts for Asians (Mi et al. 2015), we used the BMI to define underweight (<18.5 kg/m^2^), normal weight (18.5–23.9 kg/m^2^), overweight (24–27.9 kg/m^2^) and obesity (≥28 kg/m^2^), with the under 18.5 category (n = 257) removed from the analysis.

The occurrence of ischemic stroke was determined by an epidemiological questionnaire and was defined as a history of cerebrovascular events, as demonstrated by either cranial CT or MR scan (within the past 24 months before inclusion).

### Statistical analysis

As described in detail previously (Yu et al. 2014; Chang et al. 2016), descriptive statistics were calculated for all variables, including continuous variables (expressed as the mean and standard deviation) and categorical variables (expressed as numbers and proportions). Differences among categories were evaluated using the *t* test, ANOVA, a non-parametric test or the χ^2^-test as appropriate. Comparisons between groups were performed using Scheffe’s method. Multivariate logistic regression analyses were conducted to identify independent associations of different weights and metabolic statuses with ischemic stroke using different models, and odds ratios (ORs) and corresponding 95 % confidence intervals (CIs) were calculated. All statistical analyses were performed using SPSS version 22.0 software, and *P* values of less than 0.05 were considered statistically significant.

## Results

### Baseline characteristics of participants according to different obesity phenotypes

A total of 11,150 participants were evaluated in this cross-sectional study. The prevalence rates of the different obesity types were 59.4 % for MHNO, 32.8 % for MUNO, 1.7 % for MHO and 6.1 % for MUO. Among the obese population, the prevalence of MHO was 21.8 %. There were significant differences in age, gender, race, education level, physical activity, annual income, current smoking status, sleep duration, systolic blood pressure, diastolic blood pressure, body mass index (BMI) waist circumference (WC), low-density lipoprotein cholesterol (LDL), high-density lipoprotein cholesterol (HLDL), triglycerides (TGs), and total cholesterol (TC) among our study groups (Table 1) (all *P* < 0.05).

**Table 1:** Baseline characteristics of the study population according to different obesity phenotypes

Variables	MHNO	MHO	MUNO	MUO	*P*
n (%)	6620 (59.4)	184 (1.7)	3666 (32.8)	680 (6.1)	
Age (year)	52.9 ± 10.7	49.7 ± 9.6	55.9 ± 10.2	52.9 ± 9.9	<0.001
Race					0.002
Han	6264 (94.6)	172 (93.5)	3517 (96.0)	632 (92.9)	
Other^a^	356 (5.4)	12 (6.5)	149 (4.0)	48 (7.1)	
Current smoking status	2584 (39.0)	33 (17.9)	1123 (30.6)	178 (26.2)	<0.001
Current drinking status	1696 (25.6)	32 (17.4)	640 (17.5)	128 (18.8)	<0.001
Sleep duration (h/d)	7.3 ± 1.7	7.6 ± 1.5	7.2 ± 1.8	7.3 ± 1.6	0.004
TC (mmol/L)	5.1 ± 1.0	5.2 ± 0.9	5.5 ± 1.2	5.5 ± 1.3	<0.001
TGs (mmol/L)	1.2 ± 0.8	1.2 ± 0.6	2.4 ± 1.9	2.5 ± 2.2	<0.001
HDL-C (mmol/L)	1.5 ± 0.4	1.4 ± 0.3	1.2 ± 0.3	1.2 ± 0.3	<0.001
LDL-C (mmol/L)	2.8 ± 0.8	3.1 ± 0.8	3.1 ± 0.9	3.3 ± 0.9	<0.001
FPG (mmol/L)	5.5 ± 1.1	5.3 ± 0.6	6.5 ± 2.1	6.5 ± 2.0	<0.001
SBP (mmHg)	135.8 ± 22.1	138.7 ± 21.7	150.1 ± 22.5	153.9 ± 22.8	<0.001
DBP (mmHg)	79.3 ± 11.1	81.8 ± 11.3	85.7 ± 11.5	88.8 ± 11.7	<0.001
Mean height	161.1 ± 8.0	157.7 ± 10.7	159.8 ± 8.3	160.4 ± 8.4	<0.001
Mean weight	60.4 ± 9.4	81.6 ± 10.2	66.1 ± 9.8	83.2 ± 10.2	<0.001
WC (cm)	78.0 ± 7.9	95.4 ± 9.2	86.6 ± 7.3	99.0 ± 7.9	<0.001
BMI (kg/m^2^)	23.2 ± 2.7	33.0 ± 4.5	25.8 ± 2.5	32.2 ± 2.2	<0.001
Diet score	2.4 ± 1.1	2.3 ± 1.0	2.2 ± 1.1	2.3 ± 1.2	<0.001
Salt intake	6.4 ± 3.8	6.1 ± 3.6	6.5 ± 3.8	6.4 ± 3.9	0.099
Educational level (%)					<0.001
Primary school or below	3080 (46.5)	80 (43.5)	2086 (56.9)	329 (48.4)	
Middle school	2919 (44.1)	86 (46.7)	1267 (34.6)	278 (40.9)	
High school or above	621 (9.4)	18 (9.8)	313 (8.5)	73 (10.7)	
Physical activity (%)					<0.001
Light	1737 (26.2)	40 (21.7)	1296 (35.4)	224 (32.9)	
Moderate	4533 (68.5)	136 (73.9)	2130 (58.1)	414 (60.9)	
Severe	350 (5.3)	8 (4.3)	240 (6.5)	42 (6.2)	
Annual income (CNY/year)					0.045
≤5000	809 (12.2)	23 (12.5)	499 (13.6)	61 (9.0)	
5000–20,000	3629 (54.8)	100 (54.3)	1959 (53.4)	391 (57.5)	
>20,000	2182 (33.0)	61 (33.2)	1208 (33.0)	228 (33.5)	

Data are expressed as the mean ± SD or as n (%)

*CNY* China Yuan (1CNY = 0.157 USD), *BMI* body mass index, *WC* waist circumference, *SBP* systolic blood pressure, *DBP* diastolic blood pressure, *FPG* fasting plasma glucose, *TC* total cholesterol, *TGs* triglycerides, *LDL*-*C* low-density lipoprotein cholesterol, *HDL*-*C* high-density lipoprotein cholesterol

### Characteristics of participants with or without ischemic stroke

The results presented in Table 2 show that the subjects with ischemic stroke were significantly older and had a significantly higher BMI, WC, systolic blood pressure, diastolic blood pressure, and fasting plasma glucose, LDL, TC, and TG levels and a lower HDL level than those without stroke (all *P* < 0.001). In addition, a significantly higher number of subjects with ischemic stroke had a lower education level, annual income and physical activity level (all *P* < 0.001). However, there were no significant differences in race, sleep duration, mean weight or salt intake.

**Table 2:** Characteristics of study participants with or without ischemic stroke

Variables	Ischemic stroke		
	Yes	No	*P*
n (%)	353 (3.2)	10,797 (96.8)	
Age (year)	63.3 ± 8.5	53.5 ± 10.5	<0.001
Race			0.443
Han	332 (94.1)	10,253 (95.0)	
Other^a^	21 (5.9)	544 (5.0)	
Current smoking status	106 (30.0)	3812 (35.3)	0.041
Current drinking status	41 (11.6)	2455 (22.7)	<0.001
Sleep duration (h/d)	7.1 ± 2.1	7.3 ± 1.7	0.122
TC (mmol/L)	5.4 ± 1.0	5.2 ± 1.1	0.001
TGs (mmol/L)	2.1 ± 1.9	1.6 ± 1,5	<0.001
HDL-C (mmol/L)	1.3 ± 0.4	1.4 ± 0.4	<0.001
LDL-C (mmol/L)	3.2 ± 0.8	2.9 ± 0.8	<0.001
FPG (mmol/L)	6.5 ± 2.1	5.9 ± 1.6	<0.001
SBP (mmHg)	160.5 ± 27.0	141.0 ± 23.0	<0.001
DBP (mmHg)	86.9 ± 11.9	81.9 ± 11.7	<0.001
Mean height	158.4 ± 8.0	160.6 ± 8.2	<0.001
Mean weight	63.9 ± 10.3	64.1 ± 11.4	0.784
WC (cm)	85.6 ± 9.4	82.3 ± 9.8	<0.001
BMI (kg/m^2^)	25.4 ± 3.6	24.8 ± 3.7	0.001
Diet score	1.9 ± 1.2	2.3 ± 1.1	<0.001
Salt intake	6.7 ± 3.9	6.4 ± 3.7	0.23
Educational level (%)			<0.001
Primary school or below	245 (69.4)	5330 (49.4)	

Data are expressed as the mean ± SD or as n (%)

*CNY* China Yuan (1CNY = 0.157 USD), *BMI* body mass index, *WC* waist circumference, *SBP* systolic blood pressure, *DBP* diastolic blood pressure, *FPG* fasting plasma glucose, *TC* total cholesterol, *TGs* triglycerides, *LDL*-*C* low-density lipoprotein cholesterol, *HDL*-*C* high-density lipoprotein cholesterol

### The prevalence of ischemic stroke according to different obesity phenotypes

**Fig. 1:**
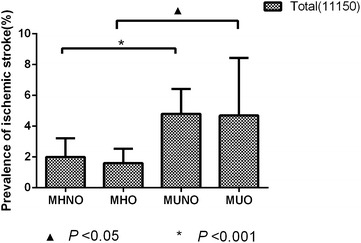
The prevalence of ischemic stroke among the total population

**Fig. 2:**
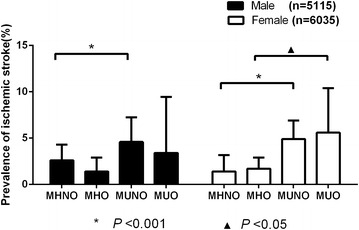
The prevalence of ischemic stroke for different groups by gender

The prevalence of ischemic stroke in our study was 3.2 % (Table 2). As shown in Fig. 1, the prevalence of ischemic stroke among the total population was significantly increased for MUNO (4.9 %) and MUO (4.8 %) and was decreased for MHNO (2.0 %) and MHO (1.7 %). As shown in Fig. 2, among the females, the MUO group had the highest prevalence of ischemic stroke (5.6 %), and the MHNO group had the lowest prevalence (1.4 %) compared with the other three groups (*p* < 0.001); however, among the males, the MUNO group had the highest prevalence of ischemic stroke (4.9 %), and the MHO group had the lowest prevalence (1.4 %) (*p* < 0.001).

### Multiple regression analyses of ischemic stroke and obesity phenotypes

As shown in Table 3, multiple regression analyses of the total population adjusted for age, race, gender, education level, annual income, current smoking status, current drinking status, diet score, salt intake, physical activity, and sleep duration showed that the subjects with MUNO and MUO were more likely to experience ischemic stroke than those with MHNO (OR 2.136, 95 % CI 1.677–2.720; OR 2.712, 95 % CI 1.798–4.092; all *p* < 0.001). However, multiple regression analyses by gender showed minor different results. Among the male population, after adjusting for the same factors mentioned above, the subjects with MUNO were more likely to experience ischemic stroke than those with MHNO (OR 1.855, 95 % CI 1.312–2.622; *p* < 0.001); MUO males also had a higher OR for ischemic stroke than MHNO males, but the difference wasn’t significant (OR 1.842, 95 % CI 0.888–3.819; *p* = 0.101); compared with MHNO, the OR for ischemic stroke in MHO males was lower, but the difference was not significant, either(OR 0.782, 95 % CI 0.100–5.642; *p* = 0.782). Among the female population, multiple regression analyses adjusted for the same factors mentioned above revealed that females with MUNO and MUO were more likely to experience ischemic stroke than females with MHNO (for MUNO, OR 2.528, 95 % CI 1.762–3.626; for MUO, OR 3.592, 95 % CI 2.117–6.094; all *p* < 0.001); in addition, the OR for ischemic stroke did not significantly differ between females with MHO and females with MHNO.

**Table 3:** Multiple logistic regression analyses for ischemic stroke

	Total		Males		Females	
	OR (95 % CI)	*p*	OR (95 % CI)	*p*	OR (95 % CI)	*p*
Grouped by Mes and obesity^a^						
MHNO	1.000 (reference)		1.000 (reference)		1.000 (reference)	
MHO	1.121 (0.348–3.615)	0.848	0.752 (0.100–5.642)	0.782	1.651 (0.389–7.012)	0.497
MUNO	2.136 (1.677–2.720)	*<0.001*	1.855 (1.312–2.622)	<0.001	2.528 (1.762–3.626)	*<0.001*
MUO	2.712 (1.798–4.092)	*<0.001*	1.842 (0.888–3.819)	0.101	3.592 (2.117–6.094)	*<0.001*
Grouped by BMI and Mes^b^						
Normal weight and no MeS	1.000 (reference)		1.000 (reference)		1.000 (reference)	
Normal weight and MeS	1.843 (0.946–3.592)	0.072	2.256 (1.269–4.011)	*0.006*	1.943 (1.289–2.930)	*0.002*
Overweight and no MeS	1.209 (0.755–1.934)	0.429	1.571 (0.817–3.022)	0.176	1.319 (0.903–1.928)	0.152
Overweight and MeS	1.975 (1.241–3.143)	*0.004*	2.920 (1.779–4.791)	*<0.001*	2.344 (1.695–3.241)	*<0.001*
Obesity and no MeS	1.559 (0.642–3.787)	0.327	1.602 (0.540–4.750)	0.395	1.510 (0.762–2.993)	0.238
Obesity and MeS	2.166 (1.282–3.659)	*0.004*	3.964 (2.373–6.621)	*<0.001*	2.898 (2.051–4.094)	*<0.001*

*OR* odds ratio, *95* *% CI* 95 % confidence interval, *MHO* metabolically healthy obesity, *MHNO* metabolically healthy non-obesity, *MUNO* metabolically unhealthy non-obesity, *MUO* metabolically unhealthy obesity, *Mes* metabolic syndrome

*p* < 0.05 was considered to be significant and typed in italics

### Results from stratification based on different BMI group

As shown in Table 4, for both males and females, people who had Mes with normal weight had higher prevalence of ischemic stroke than those who were normal weight but did not have Mes (both *p* < 0.05). Similarly, For all the participants, people who had Mes with overweight had higher prevalence of ischemic stroke than those who were overweight without (both *p* < 0.05). The female participants with obesity and Mes had significantly higher OR for ischemic stroke than those who were obesity without Mes (*p* < 0.05). The male participants with obesity and Mes had higher OR for ischemic stroke than those who were obesity without Mes, but the difference was not significantly.

**Table 4:** Prevalence of ischemic stroke according to different BMI group by Mes or not

	Prevalence of ischemic stroke		
	no Mes	Mes	*P*
*Total*			
Normal weight	73 (1.9 %)	41 (5.1 %)	<0.001
Overweight	48 (2.2 %)	95 (4.8 %)	<0.001
Obesity	10 (2.1 %)	73 (5.0 %)	0.005
*Male*			
Normal weight	50 (2.6 %)	13 (5.6 %)	0.009
Overweight	32 (2.7 %)	34 (4.6 %)	0.022
Obesity	6 (2.7 %)	24 (3.9 %)	0.384
*Female*			
Normal weight	23 (1.3 %)	28 (4.8 %)	<0.001
Overweight	16 (1.6 %)	61 (4.9 %)	<0.001
Obesity	4 (1.5 %)	49 (5.8 %)	0.005

*Mes* Metabolic syndrome

As shown in Table 3, compared with those who were normal weight without MeS, after adjusting for the same factors mentioned above, participants who had Mes with overweight or obesity had significantly higher OR for ischemic stroke (all *p* < 0.05); Participants who didn’t have Mes in overweight or obesity also higher OR for ischemic stroke, but the difference was not significant.

## Discussion

The main finding of this study was that individuals with MUO or MUNO were more likely to experience ischemic stroke than those with MHNO, and differences were observed between the genders.

This is the first study to investigate the prevalence rates of ischemic stroke among different obesity phenotypes using the ATP-III criteria for defining metabolic health. Previous studies have examined the associations of different obesity phenotypes with diabetes and CVD. Researchers reported that compared with metabolically healthy, normal-weight individuals, MHO individuals are more likely to have metabolic risk factors and incident diabetes but not CVD/stroke after several years of follow-up (Appleton et al. 2013). In our study, we similarly found that MHO was not associated with an increased prevalence of ischemic stroke; however, Sarah did not analyze the other obesity phenotypes, such as MUO. Hinnouho et al. analyzed the risks of cardiovascular disease and type 2 diabetes in individuals with different body mass indexes and metabolic health statuses. They reported that the MHO phenotype is associated with lower risk of type 2 diabetes than the metabolically unhealthy obese phenotype but that the risk of CVD is equally elevated for both obesity phenotypes (Hinnouho et al. 2015). In our study, we obtained different results, finding that not only MUO but also MUNO were associated with an increased prevalence of ischemic stroke among the total population. However, Hinnouho et al. did not conduct separate subgroup analysis of ischemic stroke.

In order to confirm the effect by Mes, we did stratification based on different BMI group. Results showed that, compared with people who were normal weight without Mes, participants who were in Mes with overweight or obesity had significantly higher OR for ischemic stroke; participants who were not in Mes with overweight or obesity did not showed OR significantly higher. These results evidenced that it was Mes increased the risk for ischemic stroke.

Previous study indicated many factors could associated with ischemic stroke such as age, smoking, excessive drinking including BMI (Long et al. 2016; Al-Rubeaan et al. 2016). However, after adjusted the related factors mentioned above, our study still confirm it was metabolic abnormalities not BMI itself increase the risk of ischemic stroke.

Some studies have examined the mechanisms of the association with CVD with obesity phenotypes. Karunakaran Indulekha et al. have analyzed the associations of adipokines and inflammatory and oxidative stress markers with obesity phenotypes. They have found that the metabolically obese phenotype is characterized by altered adipokine and inflammatory profiles, which could indicate that individuals with this phenotype are at high risk of type 2 diabetes mellitus and cardiovascular disease (Karunakaran et al. 2015). Scott Ahl et al. have reported that ADPN is associated with better metabolic health in both non-obese and obese white individuals. They have concluded that ADPN and peripheral adiposity play key roles in determining metabolic health, independent of BMI (Ahl et al. 2015). However, the mechanisms underlying the relationships between stroke and the different obesity phenotypes require further targeted research.

In addition, there is a novel result of our study. Among the females, the MUO group had the highest prevalence of ischemic stroke, whereas among the males, the MUNO group had the highest prevalence. Moreover, multiple regression analyses by gender showed that ischemic stroke was more likely to affect the males with MUNO, whereas it was more likely to affect the females with MUNO or MUO. These results indicate that metabolism and not obesity itself causes CVD, in agreement with other studies (Millan-Nunez et al. 2016; Stojanović et al. 2015; Turker et al. 2015). However, the differing results according to gender require further investigation. According to previous studies, the effects of hormones should be considered because estrogen in women is known to be associated with adipocytes function (Frankenfeld et al. 2004; Tworoger et al. 2004; Luo et al. 2003).

Our study has some limitations. First, it is a cross-sectional study; thus, we could not distinguish between cause and effect, and further longitudinal study should be conducted to verify our conclusions. Second, the definition for obesity and unhealthy metabolism was based on criteria for Asians by WHO and ATP-III components respectively, so they could not be representative globally. Finally, we used BMI as a criterion for obesity, but BMI only describes overall body mass and does not distinguish between fat and muscle or differentiate among different fat distributions. We also used WC, which is a better representation of metabolic abnormalities, as an indicator of obesity-related metabolic abnormalities.

## Conclusions

Our findings indicate that ischemic stroke is likely related to poor metabolic health rather than to obesity itself. Thus, controlling metabolic abnormalities may be an effective way to decrease the risk of ischemic stroke. Furthermore, particularly for males, greater consideration should be given to those in the metabolically unhealthy non-obesity category.
